# Macrophage polarization in periodontal ligament stem cells enhanced periodontal regeneration

**DOI:** 10.1186/s13287-019-1409-4

**Published:** 2019-11-15

**Authors:** Jiaying Liu, Bin Chen, Jun Bao, Yangheng Zhang, Lang Lei, Fuhua Yan

**Affiliations:** 1 Department of Periodontology, Nanjing Stomatological Hospital, Medical School of Nanjing University, Nanjing, 210008 China; 2 Department of Orthodontics, Nanjing Stomatological Hospital, Medical School of Nanjing University, Nanjing, 210008 China

**Keywords:** Periodontal ligament stem cells, Macrophage polarization, Periodontal regeneration

## Abstract

**Background:**

The inflammation and regeneration process may be accompanied by the shift in the M1/M2 polarization of macrophages to adapt to extracellular signals. How the macrophages responded to the altered immunological environment in the periodontal niche after stem cell transplantation has never been explored. The purpose of present study is to investigate whether M1/M2 polarization of macrophages participated in the tissue homeostasis and wound healing during periodontal ligament stem cell (PDLSC)-based periodontal regeneration.

**Methods:**

A rat periodontal defect model was utilized to observe the regeneration process in the PDLSC transplantation-enhanced periodontal repair. Dynamic changes in the markers of M1/M2 macrophages were observed on days 3, 7, and 21 post surgery. In addition, the outcome of regeneration was analyzed on day 21 after surgery. To further investigate the effect of PDLSCs on macrophage polarization, the conditioned medium of PDLSCs was utilized to treat M0, M1, and M2 macrophages for 24 h; markers of M1/M2 polarization were evaluated in macrophages.

**Results:**

Elevated bone volume and average thickness of bone trabecular was observed in the PDLSC-treated group by micro-computed tomography on day 21. In addition, enhanced periodontal regeneration was observed in the PDLSC-treated group with cementum-like structure regeneration and collagen fiber formation, which inserted into the newly formed cementum. On day 3, PDLSC transplantation increased IL-10 level in the periodontal tissue, while decreased TNF-α in the early stage of periodontal regeneration. On day 7, enhanced CD163+ cell infiltration and heightened expression of markers of M2 macrophages were observed. Furthermore, conditioned medium from PDLSC culture induced macrophage polarization towards the anti-inflammatory phenotype by downregulating TNF-α and upregulating IL-10, Arg-1, and CD163 in vitro.

**Conclusions:**

PDLSCs could induce macrophage polarization towards the M2 phenotype, and the shift in the polarization towards M2 macrophages in the early stage of tissue repair contributed to the enhanced periodontal regeneration after stem cell transplantation. Therefore, signals from the transplanted PDLSCs might alter the immune microenvironment to enhance periodontal regeneration.

## Background

Periodontitis, a chronic inflammatory disease affecting the supporting tissue around the teeth, not only has a negative effect on individuals’ oral function, but also has been implicated in the progress of several systemic diseases, including diabetes mellitus, cardiovascular diseases, and Alzheimer’s disease [1–4]. Despite its widespread prevalence in the general population, especially the senile persons, traditional periodontal regenerative therapies, including enamel matrix derivative (EMDs) application and bone graft, achieved only limited success in the intrabony periodontal defects [5].

The structural and interactive complexity of periodontal tissue is considered to be one of the reasons why it is difficult to regenerate periodontium [5]. Among various mesenchymal stem cells (MSCs) used for periodontal regeneration, PDLSCs were proved to be a reliable source to form new cementum-like structure in vivo [6]. In recent preclinical studies, treatment based on PDLSCs showed promising results in tissue regeneration [7, 8]. Due to their availability and feasibility of harvest during tooth extraction, PDLSCs may become an ample source of stem cells, though limitation still exists [9]. A latest randomized clinical trial testified the clinical efficacy of PDLSCs, but results about clinical parameters showed no significance compared to the control group (with bone filling material only) [9].

The paracrine of MSCs that regulate local microenvironment was considered to be one of the major mechanisms of how it might improve regeneration outcome [10]. Immune cells, including monocytes and macrophages, are an integral part of the regenerative process by sensing the extracellular signals, such as hypoxia, ischemia, and MSCs [10]. Therefore, interaction between local stem cells and immune cells in the periodontal microenvironment may modify the regenerative process [11]. Such phenomenon was found in multiple tissues, such as the bone, muscle, and central nerve system [12–14].

Macrophages are characterized by its plasticity that is commonly divided into classic-activated macrophage (M1) and alternatively activated macrophage (M2) [15–17]. Although different phenotypes of macrophages exert pro-inflammatory, anti-inflammatory, and even other undiscovered functions, their presence in a well-organized order is indispensable for ideal tissue reconstruction [18]. A research found depletion of macrophages eliminated the beneficial effect of transplanted MSCs, and indicated macrophages could be the primary target of MSCs’ immunomodulation [19]. According to current studies, it plays a critical role in microenvironment homeostasis and is a determinant factor in tissue repair and regeneration [16, 20, 21].

The unique complicated periodontal microenvironment could provide a distinctive interface to explore the interaction between PDLSCs and macrophages during periodontal regeneration. Although the stem cell-based treatment has been widely investigated with both systemic and local application, the mechanism of periodontal regeneration enhanced by PDLSCs is not fully explored. Whether and how macrophages may participate in the periodontal regeneration remained unknown. Therefore, the aim of our research was to observe the dynamic changes of macrophage polarization after PDLSC transplantation. We found that the early increased M2 polarization of macrophages by stem cell transplantation may account for the enhanced periodontal regeneration.

## Materials and methods

### Isolation, culture, and identification of PDLSCs

Five-week male Sprague-Dawley (SD) rats were raised for PDLSCs. Animal care and experiments were performed following the institutional protocol approved by Medical School of Nanjing University.

First and second molars of SD rats were extracted for PDLSC isolation. Gingivae were carefully removed, and periodontal ligaments were digested in a solution of 3 mg/mL collagenase type I and 4 mg/mL dispase II for 1 h at 37 °C as described previously [6]. Single-cell suspensions were attained by passing through a 70-μm strainer and were seeded in a low density (10^3^–10^4^ cells) into 10-cm culture dishes using Dulbecco’s modified Eagle medium (DMEM, Gibco, USA) supplemented with 10% fetal bovine serum (FBS, Gibco, USA) and 1% penicillin/streptomycin (P/S, Hyclone, USA).

After 10-day culture, cells were fixed with paraformaldehyde (Servicebio, China) and stained with methylene blue (Sigma-Aldrich) for colony identification. Aggregates of no less than 50 cells were considered as colonies. Passages between 2 and 5 were used for the following experiments.

Osteogenic differentiation was induced as reported previously [22] and was detected by staining with Alizarin red S (Cyagen, USA). Adipogenic differentiation was conducted with 1 μM dexamethasone (Sigma-Aldrich), 0.5 mM 3-isobutyl-1-methylxanthine (Sigma-Aldrich), 200 μM Indomethacin (Sigma-Aldrich), and 10 mg/L insulin (Gibco, USA) for 5–7 days. Lipid droplets were fixed by 4% paraformaldehyde and identified by staining with Oil Red O (Sigma-Aldrich).

When the PDLSCs reached certain confluence (60–70%), fresh complete medium was changed and was collected after 24-h culture. The collected medium was centrifuged at 3000 rounds/min for 10 min to remove debris and was used as conditioned medium of PDLSCs (PDLSC-CM) [23]. Control medium was collected following same procedures without PDLSCs.

### Isolation, culture, and polarization of bone marrow-derived macrophages (BMDMs)

Femurs and tibias of SD rats were used for the isolation of BMDMs. The isolated cells were cultured using DMEM supplemented by 10% FBS, 1% P/S, and 40 ng/ml macrophage colony stimulating factor (M-CSF, PeproTech, USA). After culture for 7 days, BMDMs were stimulated for 24 h by 50 ng/ml lipopolysaccharide (LPS, Sigma-Aldrich) + 30 ng/ml interferon-γ (IFN-γ, PeproTech, USA), or 20 ng/ml interleukin-4 (IL-4, PeproTech, USA), respectively.

For conditioned culture, medium was changed by 50% of PDLSC-CM and 50% of fresh complete medium for test groups and cultured for 24 h.

### Flow cytometry

Briefly, PDLSCs were digested and stained against rat CD11b/c (FITC-conjugated; BioLegend, USA), rat CD45 (Alexa Fluor 647-conjugated; BioLegend, USA), anti-rat CD29 (PE-conjugated; BioLegend, USA), rat CD90 (PE-conjugated; BioLegend, USA), rat CD146 (PE-conjugated; Miltenyi Biotec, Germany), and human STRO-1 (Alexa Fluor-conjugated; BioLegend, USA) for cell identification. Macrophages seeded and treated in 6-well plates were digested and stained against rat CD11b/c (FITC-conjugated; BioLegend, USA) and rat CD163 (Alexa Fluor® 647-conjugated; Bio-Rad, USA) following the same protocol.

### Enzyme-linked immunosorbent assay (ELISA)

After BMDMs were stimulated to polarize for 24 h as previously described, the supernatant was collected and centrifuged at 3000 rpm/min for 10 min to remove dead cells and debris. ELISA analyses for tumor necrosis factor-α (TNF-α, Neobioscience, China) were then performed following the manufacturer’s instructions.

### RNA preparation and quantitative real-time polymerase chain reaction (q-PCR)

Total RNA was obtained by TRIzol Reagent (Thermo Fisher Scientific, USA). Complementary DNAs were prepared from total RNA by reverse transcription (PrimeScript RT Reagent Kit, Takara Bio, Japan). The primers (Table 1) for q-PCR, including GAPDH, IL-10, TNF-α, Arg-1, and iNOS, were synthesized by Genscript (China). Relative quantification was achieved using the comparative 2^−ΔΔCt^ method.

**Table 1:** Primer sequences

Primer name	Forward primer sequence (5′-3′)	Reverse primer sequence (5′-3′)
GAPDH	AATGCKTCCTGYACCACCAACTGC	TTAGCCAWATTCRTTGTCRTACCAGG
IL-10	GAGAGAAGCTGAAGACCCTCTG	TCATTCATGGCCTTGTAGACAC
TNF-α	TCTCAAAACTCGAGTGACAAGC	GGTTGTCTTTGAGATCCATGC
Arg-1	AAGAAAAGGCCGATTCACCT	CACCTCCTCTGCTGTCTTCC
iNOS	CTCACTGTGGCTGTGGTCACCTA	GGGTCTTCGGGCTTCAGGTTA

### Western blot

The method of western blot was described previously [22]. Primary antibodies used for the procedure included iNOS (Novus, USA), Arg-1(Proteintech, USA), and β-actin (CMCTAG, USA). The images were detected using Tanon-5200 chemiluminescent imaging system (Tanon, Shanghai, China).

### Animal models and operation process

Forty-two seven-week-old SD rats were purchased from Shanghai Sino-British SIPPR/BK Lab. Animal Ltd. and were housed under specific pathogen-free conditions with a 12-h light-dark cycle for 1 week. The temperature was controlled between 20 and 26 °C with 40–70% relative humidity. Animal care and experiments were performed following the institutional protocol approved by Medical School of Nanjing University. SD rats were randomly divided into 3 groups which were as follows: (1) Blank: defects only; (2) Membrane: defects treated with membrane only; and (3) PDLSCs: defects treated with PDLSCs carried by membrane. For the PDLSC group, 20 μl PDLSCs suspension (10^6^ cell/ml) was added on to the membrane (Bio-Gide®, Geistlich, Switzerland) that was trimmed to a size of 5 mm × 2 mm and put in 12-well plates. More complete medium was supplemented into the wells to 500 μl/well 30 min later. PDLSCs seeded onto the membrane were cultured overnight.

For the operation, briefly, the anesthesia was carried out by intra-peritoneal injection of pentobarbital sodium (Sigma-Aldrich) at a dose of 60 mg/kg. The incision was made on the skin of the right orofacial side in an anterior-posterior direction to gain access to the mandible. Defect was formed using a low-speed dental drill with profuse irrigation of saline till the roots of first and second molars were exposed. The dimension of the defect was approximately 5 mm × 1.5 mm × 0.8 mm. The wound was carefully irrigated and treated as previously mentioned. Finally, the incision was sutured. All animals received injection of penicillin after surgery for 3 days.

Three and 7 days after operation, four animals were randomly sacrificed in each group. Twenty-one days after operation, six animals were randomly sacrificed in each group. The right mandibles were harvested and fixed with 4% paraformaldehyde for further procedures.

### Scanning electron microscopy

Topographical features of the collagen membrane and seeded PDLSCs were examined by scanning electron microscopy (SEM) (SU8010, Hitachi, Japan). PDLSC seeding was described above. The complex was washed gently with PBS, then was fixed in 2% glutaraldehyde overnight at 4 °C. Samples were then dehydrated by rinsing in ethanol solution with series of concentration (from 30% to 100%, v/v) for 15 min at each step. Once dried and gold-sprayed, the samples were examined via SEM.

### Micro-computed tomography scanning

All samples were scanned using a micro-computed tomography (Micro-CT) machine (Hiscan XM Micro CT, Suzhou Housefield Information Technology Co. Ltd., China) with an acquisition protocol (60 kV, 134 μA and 0.5-mm voxel size). The files were imported into Dataviewer (Bruker, USA) and reconstructed for analysis. The tissue volume selected for quantification analysis was fixed in the shape and size and was in the same area for each sample to minimize error.

### HE staining and immunostaining

Specimens were fixed with 4% paraformaldehyde and decalcified (EDTA, Servicebio, China) for 5–6 weeks at room temperature, then dehydrated. Histological sections were cut buccolingually for HE (Servicebio, China), Masson trichrome (Servicebio, China) and immunohistochemistry staining. Antibodies against inducible nitric oxide synthase (iNOS, Servicebio, China), CD163 (Servicebio, China), CD206 (Abcam, USA), TNF-α (Servicebio, China), and IL-10 (Servicebio, China) were used in the present experiment.

Sections were scanned using Pannoramic MIDI (3DHISTECH Ltd., Budapest, Hungary) and were browsed with CaseViewer software (3DHISTECH Ltd., Budapest, Hungary). For sections of every sample, pictures of certain area were captured by CaseViewer and were used for analysis. The number of iNOS+, CD163+, and CD206+ cells were counted manually in the fixed size region whose location was selected randomly in the captured field (ImageJ). For IL-10 and TNF-α staining, the positively stained area in the field was identified and quantified using preset constant value of color threshold (ImageJ).

### Statistical analysis

All data were expressed as mean ± standard deviation. The data obtained in vitro were from at least three independent experiments. Differences were analyzed by two-tailed unpaired Student’s *t* test or one-way ANOVA as appropriate. A significance level of less than 0.05 was adopted.

## Results

### Identification of PDLSCs

**Fig. 1:**
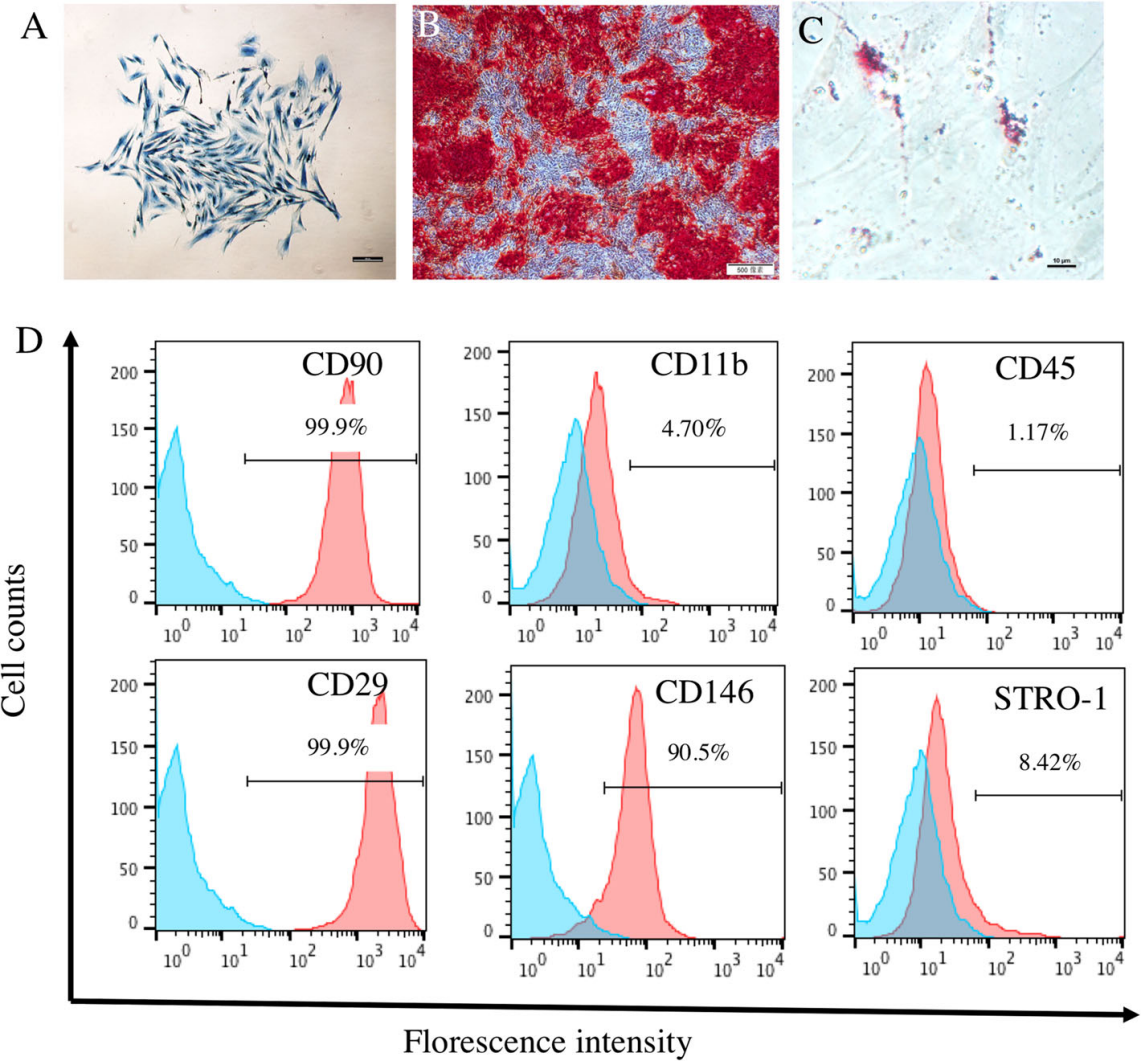
Identification of PDLSCs. **a** Single colony formation can be observed after cells were plated in a low density and were stained with methylene blue. **b** Osteogenic differentiation of PDLSCs. **c** Adipogenic differentiation of PDLSCs. **d** The expression of MSC-related surface markers of PDLSCs. Scale bar 100 μm (**a**), 500 μm (**b**), and 10 μm (**c**)

Cells derived from rat periodontal ligament (PDL) were capable of forming single colony after culture for 10 days in low density (Fig. 1a). In addition, harvested cells displayed osteogenic and adipogenic capability as shown by positive staining of alizarin red after 21-day induction and Oil red O staining after 5–7 days of induction respectively (Fig. 1b). The expression of surface markers, including CD90, CD11b, CD45, CD29, CD146, and STRO-1, was consistent with the pattern of MSCs (Fig. 1d). Therefore, we considered the group of cells isolated from PDL tissue met the basic criteria of MSCs, and the harvested cells were PDLSCs [24].

### PDLSCs promoted periodontal regeneration in vivo

**Fig. 2:**
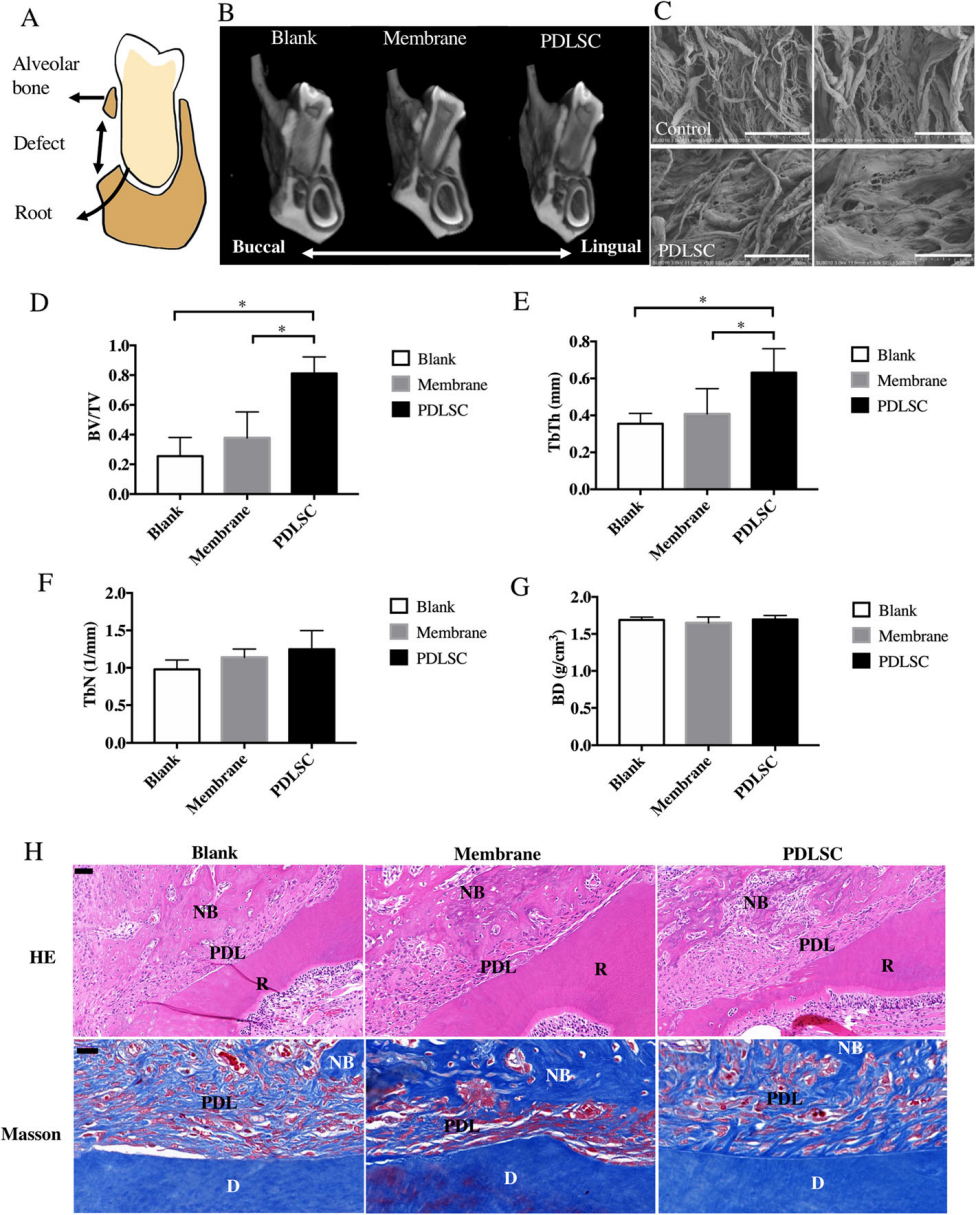
PDLSCs promoted periodontal regeneration in vivo on day 21. **a** Schematic diagram of periodontal defect model. **b** Reconstruction images of micro-CT showed enhanced regeneration of alveolar bone with transplanted PDLSCs. **c** Scanning electron microscope showed PDLSCs adhered to the collagen membrane after culture overnight. Scale bar: 100 μm (Left), 30 μm (Right). **d**–**g** Parameters to evaluate new bone: bone volume/tissue volume (**d**), average thickness of bone trabecula (**e**), average number of bone trabecula (**f**), and bone density (**g**). **h** HE and Masson staining of histological sections. Scale bar 50 μm (HE), 20 μm (Masson). NB, new bone; PDL, periodontal ligament; R, tooth root. *p < 0.05

Allogeneic PDLSCs carried by membrane materials were transplanted into the periodontal defect in rats (as illustrated in Fig. 2a, c). The reconstructed images of Micro-CT indicated relatively more continuous bone regeneration in the defect area in the PDLSC-treated group at 21 days after surgery, whereas the bone defect was more apparent in the other 2 groups (Fig. 2b). Significantly, more bone volume and average thickness of bone trabecular (TbTh) was observed in the stem cell-transplantation group (Fig. 2d, e), whereas no significant difference in average number of bone trabecular (TbN) and bone density (BD) was found (Fig. 2f, g). Similar to micro-CT data, the newly regenerated bone almost completely covered the periodontal defect in the PDLSC group (Fig. 2h). Moreover, cementum-like structures were identified in the PDLSC group (Fig. 2h). Collagen fibers were well oriented and inserted into cementum-like structure formed on dentine surface, indicating cementum regeneration, one critical event in the periodontal regeneration. However, less similar structures can be observed in other 2 groups. Therefore, our experiment suggested that the cementum regeneration might be related to the transplanted PDLSCs.

### Effect of PDLSCs on macrophage polarization and cytokine secretion in vivo

**Fig. 3:**
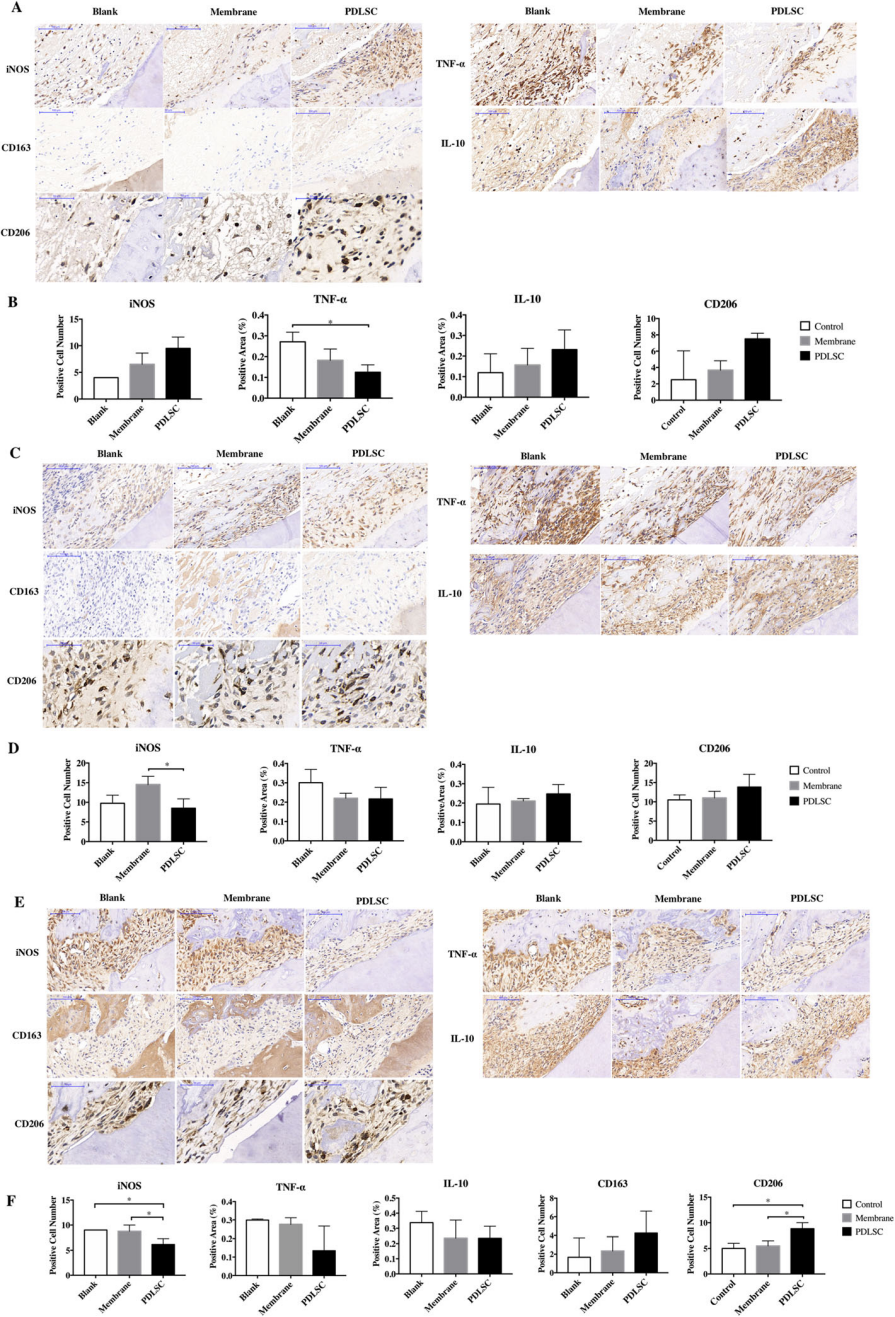
Effect of PDLSCs’ transplantation on macrophage polarization and cytokine secretion. The immunochemistry staining against CD163, CD206, iNOS, TNF-α, and IL-10 on day 3 (**a**), day 7 (**c**), and day 21 (**e**) after surgery. Quantification of the number of iNOS+ cells, the number of CD163+ cells and CD206+ cells, and the positive area of TNF-α and IL-10 in the field was performed on day 3 (**b**), day 7 (**d**), and day 21 (**f**) after surgery. *p < 0.05

Since transplanted PDLSCs promoted periodontal regeneration, we subsequently examined whether PDLSCs might affect inflammation environment through periodontal healing, in terms of cytokine secretion and macrophage polarization. Three days after surgery, a higher level of IL-10 and a significantly lower level of TNF-α secretion was observed in the PDLSC group when compared to the other 2 groups (Fig. 3a, b). The level of TNF-α in the PDLSC group remained to be the lowest among groups on day 7 and day 21 (Fig. 3c–f). IL-10 secretion level was stable in the PDLSC group from days 3–21, but had been increasing in the Blank and Membrane groups from days 7–21. Thus, IL-10 in the PDLSC group became lower compared to other groups on day 21, at late stage of healing (Fig. 3a–f). Overall, in vivo, it exerted anti-inflammatory function that lowered TNF-α through the process and enhanced IL-10 secretion at early stage of healing.

Immunostaining against M1 marker iNOS revealed larger amount of iNOS^+^ cells in the PDLSC group (Fig. 3a, b) on day 3. The number of iNOS+ cells was then decreased and became the least on day 21 compared to other groups. PDLSCs seemed to induce iNOS+ cells at early stage and then turned to inhibit as healing went along. No CD163+ cells were detected in any groups on day 3. However, on day 7, a few CD163+ cells were observed in the PDLSC group but not in others. CD163+ cells could be seen in all groups on day 21, but larger number of CD163+ cells were found in the PDLSC group (Fig. 3c, e–f). CD206+ cells were observed in all groups since day 3, though no difference was detected except for day 21 (Fig. 3a–f). The number of CD206+ cells in the PDLSC group was significantly larger than in the control and Membrane groups on day 21 (Fig. 3e, f). According to our results, PDLSCs induced CD163+ cells since day 7 to day 21 and CD206+ cells on day 21. The series of changes of inflammatory response triggered by PDLSCs were observed in the process of periodontal regeneration ended with superior outcome.

### Macrophage polarization and identification

**Fig. 4:**
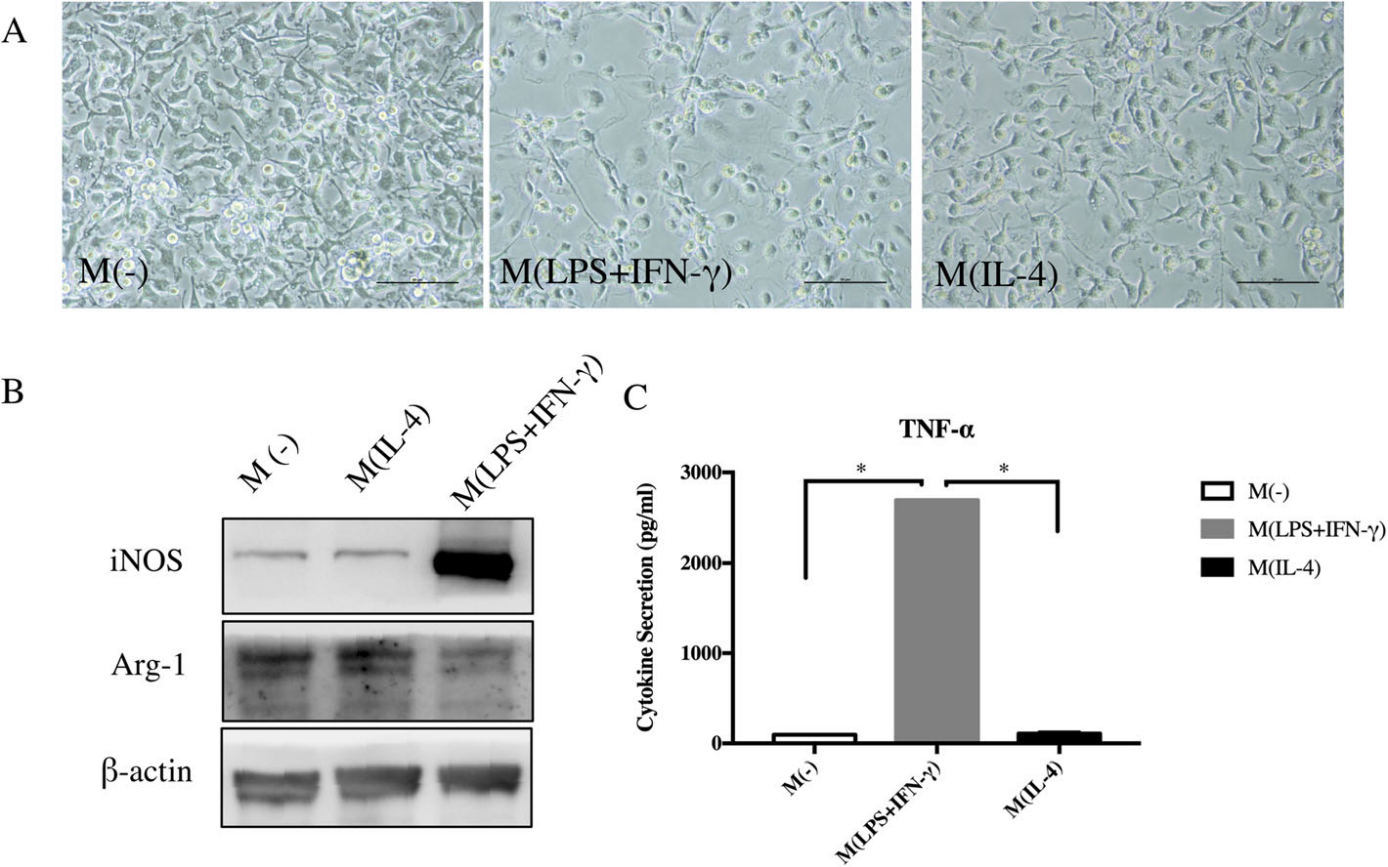
Macrophage polarization induced by LPS+IFN-γ/IL-4. **a** Polarized macrophages displayed different morphologies. **b** Western blot of iNOS, Arg-1, and β-actin of control expressed by macrophages from different treated groups. **c** TNF-α secretion of macrophages in each group. Scale bar 50 μm (**a**). M(-), non-activated macrophages. *p < 0.05

Known that specific macrophage phenotype is necessary for tissue regeneration and PDLSC transplantation impacted macrophage polarization in vivo, we further investigated effects of PDLSCs on subsets of macrophages in vitro. Macrophages stimulated by LPS and IFN-γ were flat and round, while those treated with IL-4 were rather elongated (Fig. 4a). M (LPS+IFN-γ) expressed high level of iNOS and TNF-α but low level of Arg-1 (Fig. 4b, c). In contrast, IL-4-treated macrophages exhibited high level of Arg-1 and low level of iNOS and TNF-α, which was similar to non-activated macrophages (Fig. 4b, c). The characteristics of macrophages treated with different stimulus such as LPS+IFN-γ or IL-4 were in accordance with reports from related studies, referring to pro-inflammatory and anti-inflammatory phenotypes respectively [17].

### PDLSCs increased the number of CD163+ cells by paracrine effect

**Fig. 5:**
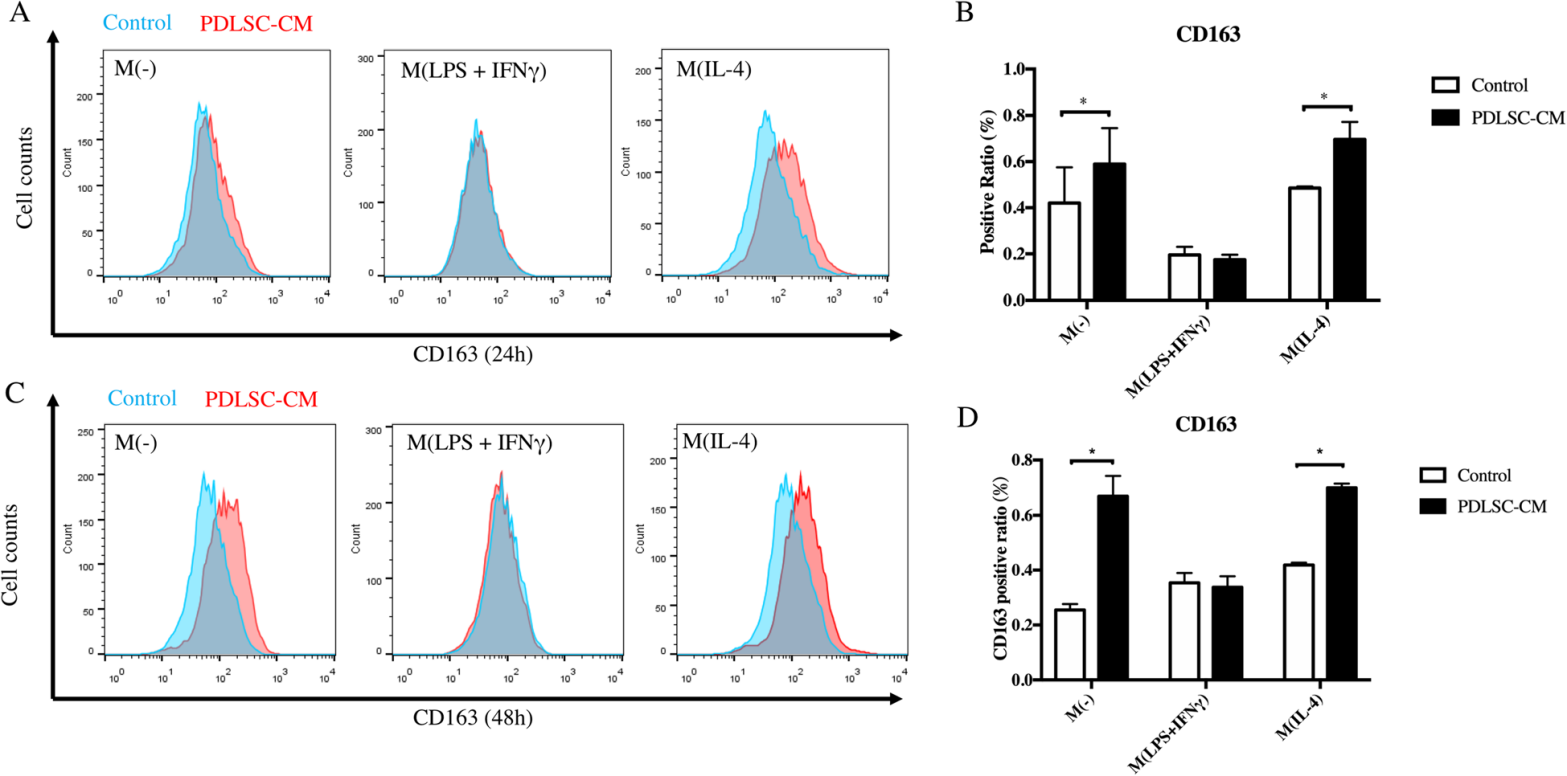
PDLSC-CM promoted the expression of CD163 in non-activated and anti-inflammatory macrophages. **a** PDLSC-CM increased CD163 expression in M(-) and M (IL-4) after 24 h treatment. **b** The quantitation and statistical analysis of **a**. **c** The effect of PDLSC-CM on CD163 expression continued after 48 h treatment. **d** The quantitation and statistical analysis of **c**. M(-), non-activated macrophages; PDLSC-CM, conditioned medium of PDLSCs. *p < 0.05

To investigate the effect of PDLSCs on macrophages, the PDLSC-CM was prepared and used to treat non-activated and polarized macrophages. CD163, a surface marker of M2 or anti-inflammatory macrophages, was detected. Both non-activated macrophages and M (IL-4) had increased proportion of CD163+ macrophages after PDLSC-CM treatment for 24 h (Fig. 5a, b). Such increased proportion of CD163+ macrophages by PDLSC-CM incubation was more pronounced at 48 h (Fig. 5c, d). However, no significant change of CD163 expression on M (LPS+IFN-γ) was found after either 24 h or 48 h of PDLSC-CM treatment. The results suggested that PDLSCs induced CD163 expression in non-activated and anti-inflammatory macrophages through its paracrine product, while having no significant effect on pro-inflammatory macrophages (Fig. 5).

**Fig. 6:**
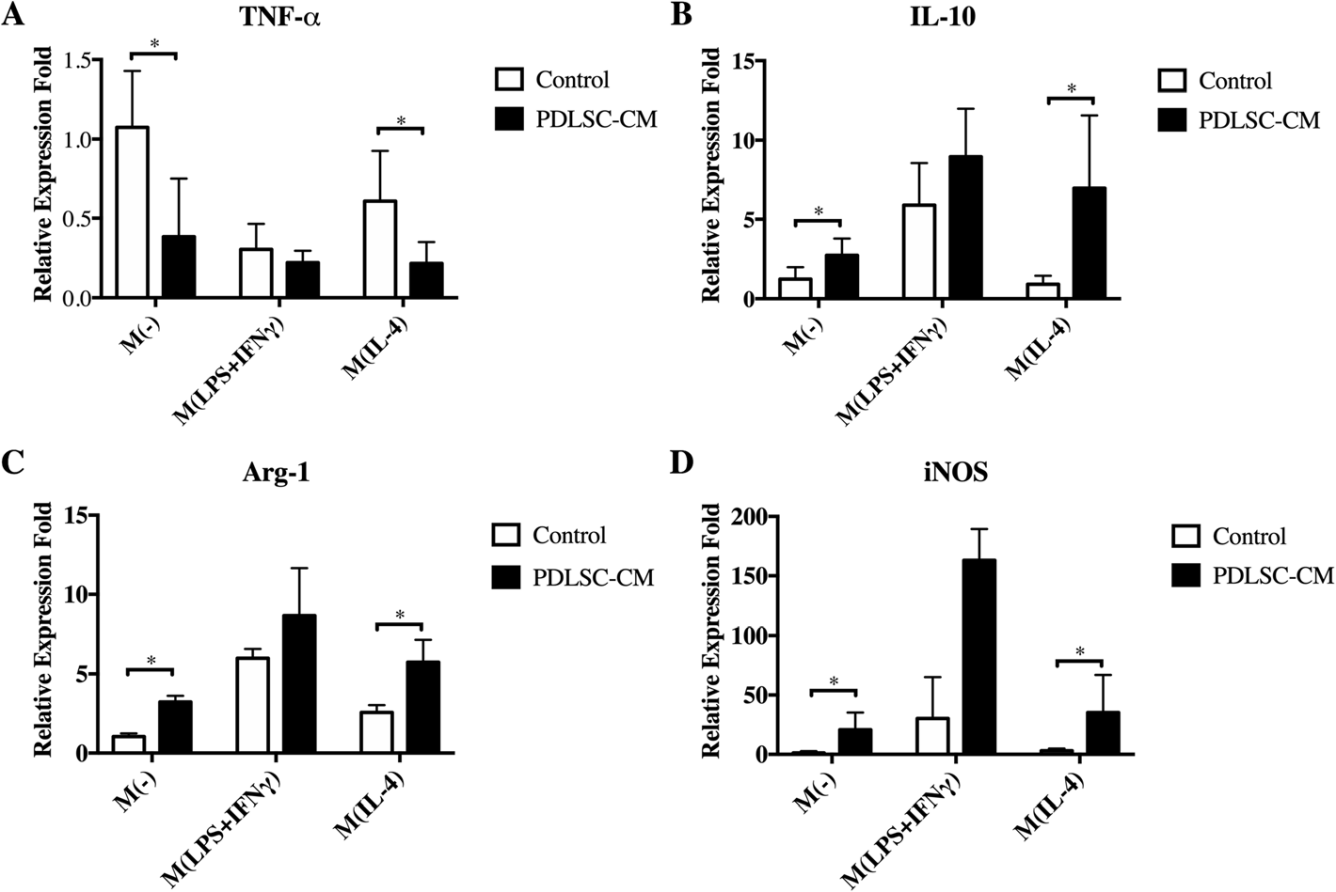
The effects of the conditioned medium (CM) derived from PDLSC cultures on the pro-/anti-inflammatory expression of different phenotypes of macrophages: **a** TNF-α, **b** IL-10, **c** Arg-1, and **d** iNOS. M(-), non-activated macrophages; PDLSC-CM, conditioned medium of PDLSCs. *p < 0.05

### In vitro anti-inflammatory function of the PDLSCs

To further explore the effect of PDLSCs on macrophages, gene transcription of TNF-α, IL-10, Arg-1, and iNOS were quantified (Fig. 6). PDLSC-CM decreased TNF-α mRNA transcription, while increased IL-10, Arg-1, and iNOS transcription in M(-) and M (IL-4). PDLSC-CM increased iNOS transcription in M (LPS+IFN-γ), while it did not alter TNF-α, IL-10, and Arg-1 transcription. Therefore, our experiment indicated that paracrine product from PDLSCs exerted anti-inflammatory effect by downregulating TNF-α and upregulating IL-10 as well as Arg-1 on non-activated and anti-inflammatory macrophages. Additionally, the intriguingly upregulated iNOS expression was also to be noted.

## Discussion

The PDLSC is one potential resource for periodontal regeneration due to its availability in the process of tooth extraction. Although the outcome of PDLSCs transplantation was reported by several pre-clinical experiments [25], our study further demonstrated that PDLSCs transplantation might improve periodontal regeneration by its paracrine effect on macrophage polarization.

In the process of tissue repair, the crosstalk between MSCs and local immune cells is crucial for achieving optimal regeneration [19]. Macrophages play an important role in the inflammatory phase of tissue repair and might be the primary target of MSC-mediated immunomodulation. Depletion of macrophages resulted in impaired bone healing [18]. In addition, macrophage deficiency eliminated the bone marrow mesenchymal stem cells’ protective effect on the LPS-induced septic shock [19]. Macrophage’s dynamic plasticity allows its elaborate regulation on tissue reconstruction [26]. Moreover, both pro-inflammatory and anti-inflammatory macrophages are critical in wound healing, and the shift in macrophage may accompany tissue repair [18]. Thus, observing the states of immune microenvironment after cell transplantation has profound implication for stem cell-based tissue repair/regeneration processes.

Our present study explored the dynamic changes of macrophages and cytokines in the periodontal healing process for the first time. We discovered that PDLSC transplantation impacted macrophage polarization, demonstrating that PDLSCs not only regulated T cells and peripheral blood mononuclear cells (PBMNCs) [27, 28], but also macrophages. According to recent studies, macrophage polarization towards anti-inflammation are always correlated with better regeneration outcome [29–31]. Stem cells from human exfoliated deciduous teeth were also reported to regulate macrophage polarization and enhance regeneration in periodontitis [31]. We found that PDLSCs reduced inflammation and induced earlier appearance of CD163+ cells on day 7 and larger number of CD163+ and CD206+ cells on day 21 in vivo, with downregulated TNF-α and iNOS at the same stage. Although both CD163 and CD206 were known as biomarkers of M2, they were involved into different cell activities [32]. Despite that, PDLSCs’ effect on CD163 and CD206 was similar in vivo. The change of immune microenvironment by PDLSC transplantation led to enhanced periodontal regeneration. The periodontal healing process would be revealed more in detail, with investigation at additional time points in further research.

Macrophages are also key effectors to osteoimmunology, where pro-inflammatory cytokines promote bone absorption, while anti-inflammatory cytokines facilitate bone formation [33]. However, how PDLSCs may regulate macrophages in periodontal healing are to be investigated. In vitro, we discovered that PDLSCs promoted non-activated and anti-inflammatory macrophages polarized further towards anti-inflammatory phenotype by downregulating TNF-α expression and upregulating IL-10 and CD163. Decreased TNF-α might lead to better healing outcome as high TNF-α may lead to impaired stem cells and reduced regeneration [34]. iNOS, a marker of M1, is also a key component to nitric oxide (NO)-dependent BMMSC-mediated immunosuppression [19, 35]. Our research further testified that iNOS was upregulated by PDLSCs in vitro and early stage of healing in vivo, which might be related to PDLSCs’ immunomodulation.

The present study suggested that immune regulation is a possible way to improve regeneration outcome. Therefore, various scaffolds that promote macrophage polarization to M2 and exert anti-inflammation effect could also be helpful if applied to periodontal defect [36–38]. Besides, our study indicated the positive effect of paracrine products from PDLSCs on macrophage polarization, though the active components are not known. The results are in accordance with a recent research that concentrated PDLSC-CM enhanced rat periodontal regeneration [39].

Periodontal wound healing is a complicated process involving multiple molecules and cell types that interact together to accomplish the regeneration process in the local environment [40]. We provided only limited information about the process and indicated several components that might participate in the process. Further studies are still needed to confirm the direct contribution of macrophages to periodontal regeneration and mechanisms behind the phenomenon. The efficacy of new approaches based on immune intervention combined with PDLSCs or its products needs to be investigated for periodontal regeneration.

## Conclusions

In summary, our research demonstrated that potentially by impacting the macrophage polarization towards M2 subtype, PDLSC transplantation could improve regeneration. Further studies are needed to clarify the mechanism of PDLSCs on macrophage polarization and its immune regulation of the periodontal micro-environment.

## Data Availability

Not applicable.

## Abbreviations

ANOVA: Analysis of variance
Arg-1: Arginase-1
BD: Bone density
BMDMs: Bone marrow-derived macrophages
BMMSCs: Bone marrow mesenchymal stem cells
DMEM: Dulbecco’s modified Eagle medium
EDTA: Ethylenediaminete traacetic acid
ELISA: Enzyme-linked immunosorbent assay
EMD: Enamel matrix derivatives
FBS: Fetal bovine serum
GAPDH: Reduced glyceraldehyde-phosphate dehydrogenase
HE: Hematoxylin and eosin
IFN-γ: Interferon-γ
IL-4: Interleukin-4
IL-10: Interleukin-10
iNOS: Inducible nitric oxide synthase
LPS: Lipopolysaccharide
M-CSF: Macrophage colony stimulating factor
M(-): Non-activated macrophages
M1: Classic-activated macrophage
M2: Alternatively activated macrophage
Micro-CT: Micro-computed tomography
MSCs: Mesenchymal stem cells
NB: New bone
P/S: Penicillin/streptomycin
PBMNCs: Peripheral blood mononuclear cells
PDL: Periodontal ligament
PDLSCs: Periodontal ligament stem cells
PDLSC-CM: Conditioned medium of periodontal ligament stem cells
q-PCR: Quantitative real-time polymerase chain reaction
R: Tooth root
SD: Sprague-Dawley
SEM: Scanning electron microscopy
TbN: Average number of bone trabecular
TbTh: Average thickness of bone trabecular
TNF-α: Tumor necrosis factor-α
